# Reconstructing Yeasts Phylogenies and Ancestors from Whole Genome Data

**DOI:** 10.1038/s41598-017-15484-5

**Published:** 2017-11-09

**Authors:** Bing Feng, Yu Lin, Lingxi Zhou, Yan Guo, Robert Friedman, Ruofan Xia, Fei Hu, Chao Liu, Jijun Tang

**Affiliations:** 1 College of Education, Zhejiang University, Hangzhou, 310028 PR China; 2 Department of Computer Science and Engineering, University of South Carolina, Columbia, SC 29208 USA; 3 College of Engineering and Computer Science, The Australian National University, Acton, ACT 2601 Australia; 4 School of Computer Science and Technology, Tianjin University, Tianjin, 300072 PR China; 5 Department of Biological Science, University of South Carolina, Columbia, SC 29208 USA; 6 Center for Quantitative Sciences, Vanderbilt University, Nashville, TN 37232 USA

## Abstract

Phylogenetic studies aim to discover evolutionary relationships and histories. These studies are based on similarities of morphological characters and molecular sequences. Currently, widely accepted phylogenetic approaches are based on multiple sequence alignments, which analyze shared gene datasets and concatenate/coalesce these results to a final phylogeny with maximum support. However, these approaches still have limitations, and often have conflicting results with each other. Reconstructing ancestral genomes helps us understand mechanisms and corresponding consequences of evolution. Most existing genome level phylogeny and ancestor reconstruction methods can only process simplified real genome datasets or simulated datasets with identical genome content, unique genome markers, and limited types of evolutionary events. Here, we provide an alternative way to resolve phylogenetic problems based on analyses of real genome data. We use phylogenetic signals from all types of genome level evolutionary events, and overcome the conflicting issues existing in traditional phylogenetic approaches. Further, we build an automated computational pipeline to reconstruct phylogenies and ancestral genomes for two high-resolution real yeast genome datasets. Comparison results with recent studies and publications show that we reconstruct very accurate and robust phylogenies and ancestors. Finally, we identify and analyze the conserved syntenic blocks among reconstructed ancestral genomes and present yeast species.

## Introduction

Phylogenetic studies used to be the domain of morphological area, and were based on outward appearances and internal structures^1^. Later, molecular characters and DNA sequencing technologies have augmented these studies in building robust phylogenies^2,3^, although they often yield conflicting results^4–8^. Because local and biased sequences may not be enough to represent the entire genome. These sequences may also evolve in distinct rates, and cause conflicting phylogenetic signals. Currently, the widely accepted phylogenetic approach to alleviate these conflicting issues is to analyze shared gene datasets, and concatenate/coalesce their results from multiple sequence alignments to obtain a final phylogeny with the maximum support^4–6^. Recently, Salichos and Rokas analyzed a yeast gene dataset with 1,070 orthologs from 23 species and discovered 1,070 phylogenies. They concatenated these results into a final phylogeny with the maximum likelihood^4^. Marcet-Houben and Vakirlis also used similar approaches to build yeast phylogenies for 19 and 34 species^5,6^. Shen *et al*. used two different methods (concatenation and coalescence) and two data matrices (amino acids or the first two codon positions), and reconstructed the 86-taxon phylogeny among the yeasts of the subphylum Saccharomycotina. In their study, 72 internodes were highly supported (14 internodes were new to their study after comparing with other publications), 11 internodes were still unresolved or equivocal^9^. Nevertheless, contradictories still exist among these studies. These conflicting phylogenies can be due to method inconsistency, compositional bias, alignment ambiguity, model misspecification, and long branches attraction^4,8^.

Genome level evolutionary events and their biological significances have been studied for 80 years^10^. Computational methods were developed in the 1990s^11,12^, and have been widely explored in phylogeny reconstructions and evolutionary mechanisms in the past three decades^13–18^. The availability of fully sequenced/annotated genomes and advanced computational algorithms have brought evolutionary studies beyond the mere sequence level^19,20^. Gene orders can be used as genome markers in genome level evolutionary studies^21^. They represent the genome content, gene permutations, and gene directions, which can reflect genome content and structural variations during evolution. Gene order based phylogeny reconstruction approaches obtain phylogenetic signals from genome level evolutionary events, and can bypass the troublesome multiple sequence alignment step in traditional methods^19,20,22^. However, gene order analyses are more computationally costly when compared with traditional sequence level phylogenetic studies, because researchers usually treat all gene order permutations for a special occasion as a single character out of billions of possible permutation states^5,16,19,23,24^.

Researchers have been working on the computational approaches for phylogeny and ancestral reconstructions on whole genome level data^13,14,16,22,24–29^. Most present approaches can only process simplified real genome datasets or simulated datasets with identical genome content and unique genome markers^14,17,18,30–33^. They are also restricted by handling complex evolutionary events, such as deletion, insertion, duplication, and whole genome duplication^14,16–18,24,31,34^. Recent phylogeny studies on real genome gene order data also had a few limitations. Luo *et al*. used the gene order data of five mammal genome to build the phylogeny, however, they only used the common shared gene orders^35^. Figueroa *et al*. and Weigert *et al*. used the gene order data from the mitochondrial genomes to study the phylogenies^36,37^. For current computational ancestral reconstruction methods, only ANGES^38^, Gapped Adjacency^26^, and MGRA2^16^ are reported to be capable of handling non-identical genome content and all types of evolutionary events. However, they still suffer from the issues of low-resolution, accuracy, and robustness. Recently, gene duplication events have also been considered in real genome ancestral reconstructions, but only for the X-chromosome of six mammals^39^.

Yeasts have been used as models for higher level complex organisms, including mammals and humans^40^. Whole-genome sequencing studies have shown that yeasts have similar genome size, gene content, and colinearity of genes along the chromosomes of species^41^. Gordon reconstructed the yeast ancestral genome that went extinct 100 million years ago with a manual approach, using the gene order data of 11 species in Yeast Genome Order Browser (YGOB) (http://ygob.ucd.ie)^42,43^. In the latest version of YGOB, Byrne and Wolfe added nine additional yeast species, and reconstructed a ‘benchmark’ version of yeast ancestral genome from 20 species using the same method^42,44^. Jean proposed a computational method to reconstruct an ancestral genome for five non-WGD (Whole-genome duplication) species in YGOB^45^. Chauve later developed a computational method to reconstruct an ancestral genome using the same dataset as Jean but with two genome marker sets^46^. The “low-resolution marker set” contains the same 135 genome makers that were used by Jean^45^. The “high-resolution marker set” contains 710 genome markers^46^. However, their studies are still based on low-resolution datasets with only five species. Even the “high-resolution set” with 710 markers is still too low to reconstruct reliable ancestral architectures for current whole genome level studies. Vakirlis *et al*. sequenced 10 non-WGD species from the Lachancea genus and developed software AnChro to reconstruct their ancestral genomes, which contained 4446 to 4799 genes^5^.

We previously developed two computational phylogeny and ancestral reconstruction approaches, MLWD (Maximum Likelihood on Whole-genome Data)^22^ and PMAG (Probabilistic Method of Ancestral Genomics)^27,32^. The previous MLWD method only had fixed evolutionary models and was also restricted in handling complex evolutionary events, such as deletion, duplication and whole genome duplication. PMAG approach could only process simulated datasets with unique genome markers and limited types of evolutionary events^27,32^. In this study, we redesigned the evolutionary models and innovated the algorithms for these two approaches. We combined these two methods to build an automated pipeline to reconstruct phylogenies and ancestral genomes for two high-resolution whole genome datasets of the Saccharomycetaceae family. Our pipeline now can process real genome data, which have non-identical genome contents, non-unique genome markers, and all types of evolutionary events, including genome rearrangements, insertion, deletion, duplication, and whole genome duplication (WGD). Finally, we identified and analyzed conserved syntenic blocks among reconstructed ancestors and present yeast species.

## Results

### Yeasts phylogenies reconstruction

**Figure 1:**
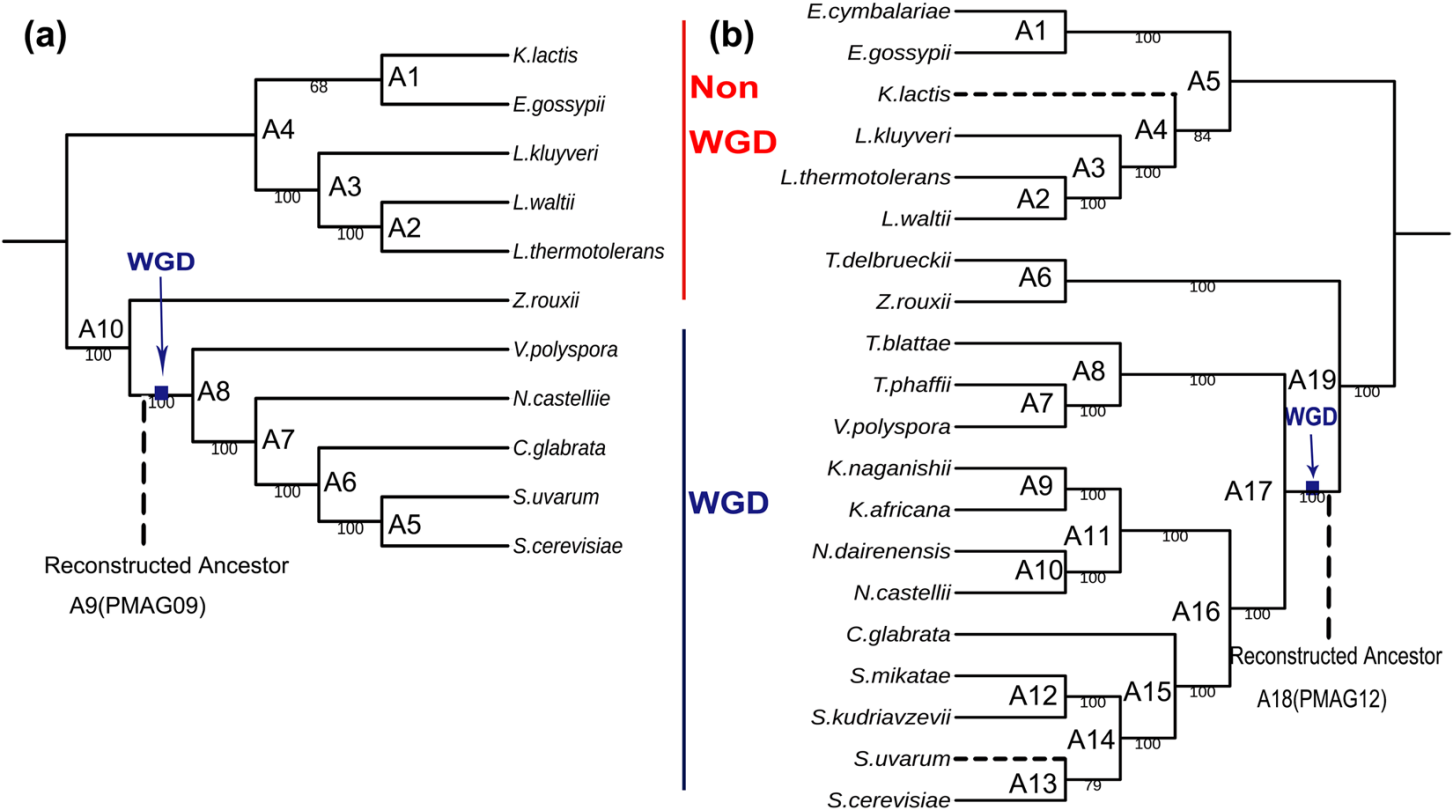
Yeasts phylogenies built from all types of evolutionary events. The red line shows the non-WGD species, while the blue line shows the post-WGD species. Each leaf represents a species, and each internal node represents a common ancestor. We mark the disagreements between these two phylogenies with dash lines. (**a**) Phylogeny built for 11 yeast species. All internal edges have bootstrapping values of 100 except the branch between Lachancea genus and Eremothecium genus, which has a bootstrapping value of 68. We label A1–A9 as the reconstructed yeast common ancestors built from the first yeast dataset. The ancestor A9(PMAG09) shows the pre-WGD ancestor PMAG09, which is in the evolutionary step before yeasts’ WGD event. The ancestor A8 is the post-WGD ancestor, had an additional genome copy from the pre-WGD ancestor A9. We also annotate the genes and their functions for all ancestral genomes in Supplementary Table 1. (**b**) Phylogeny built for 20 yeast species. All of the internal edges have a bootstrapping value of 100 except the branch between Lachancea genus and Eremothecium genus (bootstrapping value of 84) and the branch between the *S*. *cerevisiae* and *S*. *uvarum* (bootstrapping value of 79). We label A1–A18 as the reconstructed common ancestors built from the second yeast dataset. The ancestor A17(PMAG12) shows the pre-WGD ancestor PMAG12, which is in the evolutionary step before yeasts’ WGD event. The ancestor A17’ is the post-WGD ancestor, had an additional genome copy from the pre-WGD ancestor A17. We also annotate the genes and their functions for all ancestral genomes in Supplementary Table 2.

We first used our improved approach MLWD (refers to the third point of the Methods) to construct the phylogeny for the first yeast genomes dataset with 11 species, which are available in YGOB (Version 3 April 2009). Five of them are post-WGD species under four genera. Six of them are non-WGD species under other four genera. This was the same dataset that used in Gordon’s ancestor reconstruction study^43^. As shown in Fig. 1(a), we correctly classified all yeast species into their corresponding genera, and also into their corresponding groups, post-WGD and non-WGD. We compared this phylogeny with the recent studies and publications. Our phylogeny agrees with NCBI taxonomy (http://www.ncbi.nlm.nih.gov/taxonomy) on all 11 species. Our phylogeny also matches the phylogeny that was used in Gordon’s ancestral reconstruction study^43^. Recently, Salichos used a sequence alignment based maximum likelihood approach to study the phylogeny of 23 yeast species by concatenation analysis of 1,070 orthologs^4^. Marcet-Houben, Vakirlis and Shen used similar methods to infer the phylogenies of 19,34 and 86 yeast species from shared orthologs and homologs^5,6,9^. Even though our approach uses gene order data and phylogenetic signals from genome level evolutionary events, our phylogeny agrees with all of these three studies on their shared 11 species.

We continued to construct the phylogeny for the second dataset with 20 species, which are available in the latest version of YGOB (version 7 August 2012)^42,44^. Twelve of them are post-WGD species under six genera. Eight of them are non-WGD species under other five genera. As shown in Fig. 1(b), we correctly classified these 20 yeast species into their corresponding genera, and also into their corresponding groups, post-WGD and non-WGD. This phylogeny also entirely agrees with the NCBI taxonomy. Although Salichos, Marcet-Houben, Vakirlis, Shen performed similar sequence alignment based approaches to reconstruct yeast phylogenies by analyzing different gene datasets, their phylogenies still had a few conflicts with each other^4–6^. Our phylogeny also had two disagreements with these studies and with the phylogeny built in Fig. 1(a). We marked these two disagreements with dash lines in the phylogeny built from 20 species in Fig. 1(b). Our phylogeny agreed with Marcet-Houben’s phylogeny on each of the shared 18 species, except for the placement of *K*. *lactis*, which was still at the same evolutionary position in both phylogenies. However, it was closer to the Lachancea genus in our phylogeny, while it was closer to the Eremothecium genus in other sequence alignment based phylogenies^4–6,9^. We could remove this small discrepancy by using the phylogenetic signals only from genome rearrangement events (as shown in Supplementary Fig. 1). Overall, Our topology shows a global good agreement with previous knowledge on yeast phylogeny, except for the branch of *S*. *uvarum* as a sister species to *S*. *cerevisiae* which is likely to be artefactual. The differences may be caused by the inconsistencies in multiple sequence alignments and inaccuracies in gene order annotations. The yeasts input genomes are not well assembled, which have irregular and fragmentary genome structures. Many genome contigs cannot be assembled or mapped back to regular chromosomes. For example, *V*. *polyspora*, *S*. *uvarum*, *S*. *mikatae and S*. *kudriavzevii* had 269, 73, 84 and 83 genome contigs (chromosomes), respectively. These short genome contigs may cause inaccurate adjacency information and result to misleading phylogenetic signals^42^. However, our pipelines still preserve all these information, because don’t want to lose any gene adjacencies and genome content information from the original datasets.

The above results illustrate that our phylogenies are as accurate as those built from NCBI taxonomy and sequence alignment based phylogenetic approaches, although we are using the gene order data and phylogenetic signals from genome level evolutionary events. Our method can alleviate these “chromosome assembly” issues in the real yeast genome data, since our phylogeny are reconstructed from the most basic gene relationships (gene adjacencies). It skips the multiple sequence alignment step, and avoids the conflicting phylogenetic signals from local and biased DNA sequences. We provide an independent and alternative way to build the phylogenies for real genome datasets, and eliminate the conflicting issues in traditional multiple sequence alignment based approaches.

### Yeast ancestral genomes reconstruction

#### Yeast ancestral genomes reconstruction from 11 yeast species

Recently, Wolfe reported that whole genome duplication (WGD) was found in the common ancestor of six genera of Saccharomycetaceae family^41^. Gordon applied a parsimony-based approach to reconstruct the common ancestral genome dating back to 100 million years ago, right before yeasts’ WGD event^43^. Gordon used a sequence alignment based phylogeny as the guide tree, and manually reconstructed the gene orders of the ancestral genome from a dataset with 11 yeast species (this also refers to the first yeast genome dataset in this study)^42,43^. The preliminary version of this manually reconstructed ancestral genome was reported as a ‘gold standard’ in Sankoff’s studies^43,47^. In this paper, we use ‘MANUAL09’ to represent this version of the manually reconstructed yeast ancestor. In 2012, Byrne and Wolfe added nine additional yeast species to YGOB. They used the same method and reconstructed the ‘benchmark’ version ancestral genome, using genome information of 20 yeast species (this also refers to the second yeast genome dataset)^42,44^. We use ‘MANUAL12’ to represent this ‘benchmark’ version ancestral genome. The MANUAL12 ancestral genome was built from a dataset that contained more comprehensive genome information of yeast species, indicating more accurate ancestral reconstructions than the ancestor built from the first dataset (MANUAL09).

In this study, we first used our improved computational approach PMAG (the fourth point of the Methods) to reconstruct the ancestral genomes for the first yeast genome dataset. We used the phylogeny that reconstructed from the same input data in Fig. 1(a) as the guide tree. It only took 20 minutes to solve this problem, and output all internal and root ancestral genomes for these 11 yeast species. There are nine ancestral genomes reconstructed and labeled as A1–A10 in the phylogeny of Fig. 1(a). Gene numbers of each ancestral genome vary between 4,841 and 5,133. Each ancestral genome is represented by a list of ancestral genes with their corresponding gene orders that shared across the whole Saccharomycetaceae family. We further annotated all of the genes and analyzed their functions for each ancestral genome in Supplementary Dataset 1. Among our reconstructed ancestors, we use ‘PMAG09’ (also labeled as A9 in Fig. 1) to represent our pre-WGD ancestor at the same evolutionary stage with MANUAL09 and MANUAL12. The post-WGD ancestor A8 had an additional copy of pre-WGD ancestor A9, therefore, it ad the same gene orders and genome information as pre-WGD ancestor A9.

**Figure 2:**
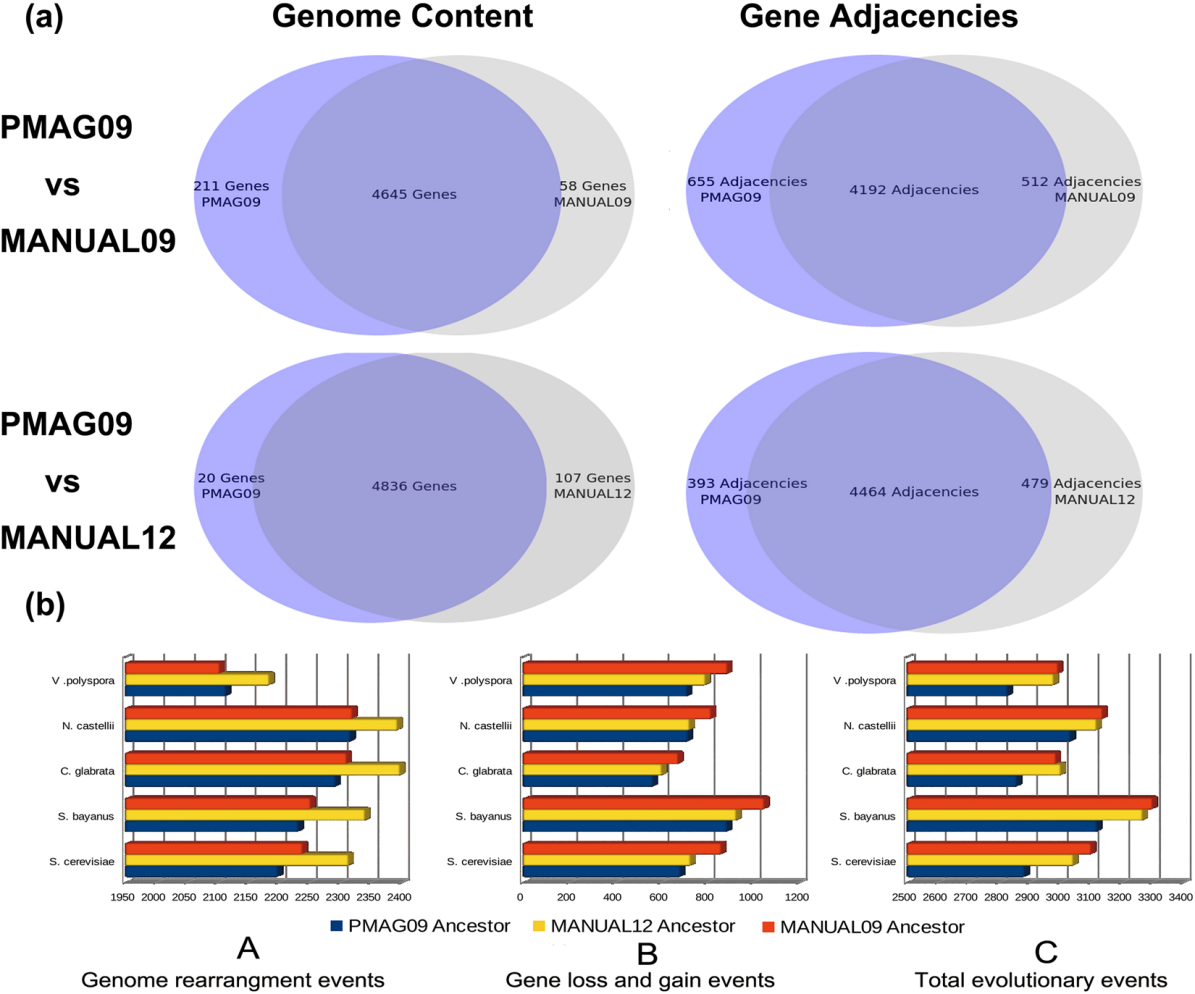
(**a**) Genome content and gene adjacency comparisons among PMAG09, MANUAL09, and MANUAL12 ancestors. (**b**) Evolutionary events comparisons among the evolutionary histories of PMAG09, MANUAL09, and MANUAL12 ancestors. Figure (**b**) (A–C) show the total number of genome rearrangements events, gene loss and gain events, and overall evolutionary events between ancestral genomes and their shared five present post-WGD descendants.

Our first results comparison was among the genome content of PMAG09, MANUAL09, and MANUAL12 ancestors, which contained 4,856, 4,703, and 4,943 genes, respectively. As Fig. 2(a) shows, the genome contents of our PMAG09 ancestor are very similar to both MANUAL09 and MANUAL12. Of all the genes in PMAG09, 4,645 (95.6%) are shared by MANUAL09, and 4,836 (99.6%) are shared by MANUAL12. We also compared all gene adjacencies among these three ancestral genomes, which could reflect the absolute differences of gene contents, directions, and permutations between any two genomes. Figure 2(a) shows that our ancestor PMAG09 shares 4,192 (86.3%) gene adjacencies with MANUAL09, and 4,464 (91.9%) gene adjacencies with MANUAL12.

We identified all gene pairs (gene adjacencies) that never split during evolution, which were called “non-split adjacencies”. There were 697 “non-split adjacencies” shared by the descendant genomes of PMAG09 and MANUAL09, and 269 “non-split adjacencies” shared by the descendant genomes of MANUAL12. in our reconstructed ancestral genomes, PMAG09 contained 638 (13.1%) “non-split adjacencies”. MANUAL09 contained 609 (12.9%) “non-split adjacencies”. MANUAL12 contained 253 (5.1%) “non-split adjacencies”. After removing the “non-split adjacencies”, PMAG09 still shared 3,554 (84.2%) adjacencies with MANUAL09 and 4,211 (91.4%) adjacencies with the “benchmark ancestral genome” MANUAL12.

We further identified and compared the number of different types of evolutionary events between the automated/manually reconstructed ancestors and their shared five present post-WGD descendant genomes. We applied a computational method to calculate genome evolutionary events based on the principle of double-cut-and-join (DCJ) operation^48,49^, which could identify all events that occurred during evolutionary history. Under DCJ operation model, if we had identified n evolutionary events between genome A and Genome B, the gene order permutations could evolve from genome A to genome B by these n events. Figure 2(b) A shows that our ancestor PAMG09 presents the least number of genome rearrangement events when compared with MANUAL09 and MANUAL12. Figure 2(b) B shows a similar result for gene loss and gain events. Likewise, Fig. 2(b) C shows that our ancestor PMAG09 has the least number of overall evolutionary events among these three ancestors.

These results demonstrate that our ancestor PMAG09 is very similar to both manually reconstructed ancestral genomes in genome content and gene adjacencies. Although our ancestor PMAG09 is reconstructed from the same data as the old version ancestor MANUAL09, it is more similar to the “benchmark” ancestor MANUAL12. MANUAL12 is built from the second dataset with more comprehensive genome information of nine additional yeast species, which indicates more accurate ancestral reconstructions. Moreover, PMAG09 has fewer evolutionary events than both MANUAL09 and MANUAL12.

#### Yeast ancestral genomes reconstruction from 20 yeast species

**Figure 3:**
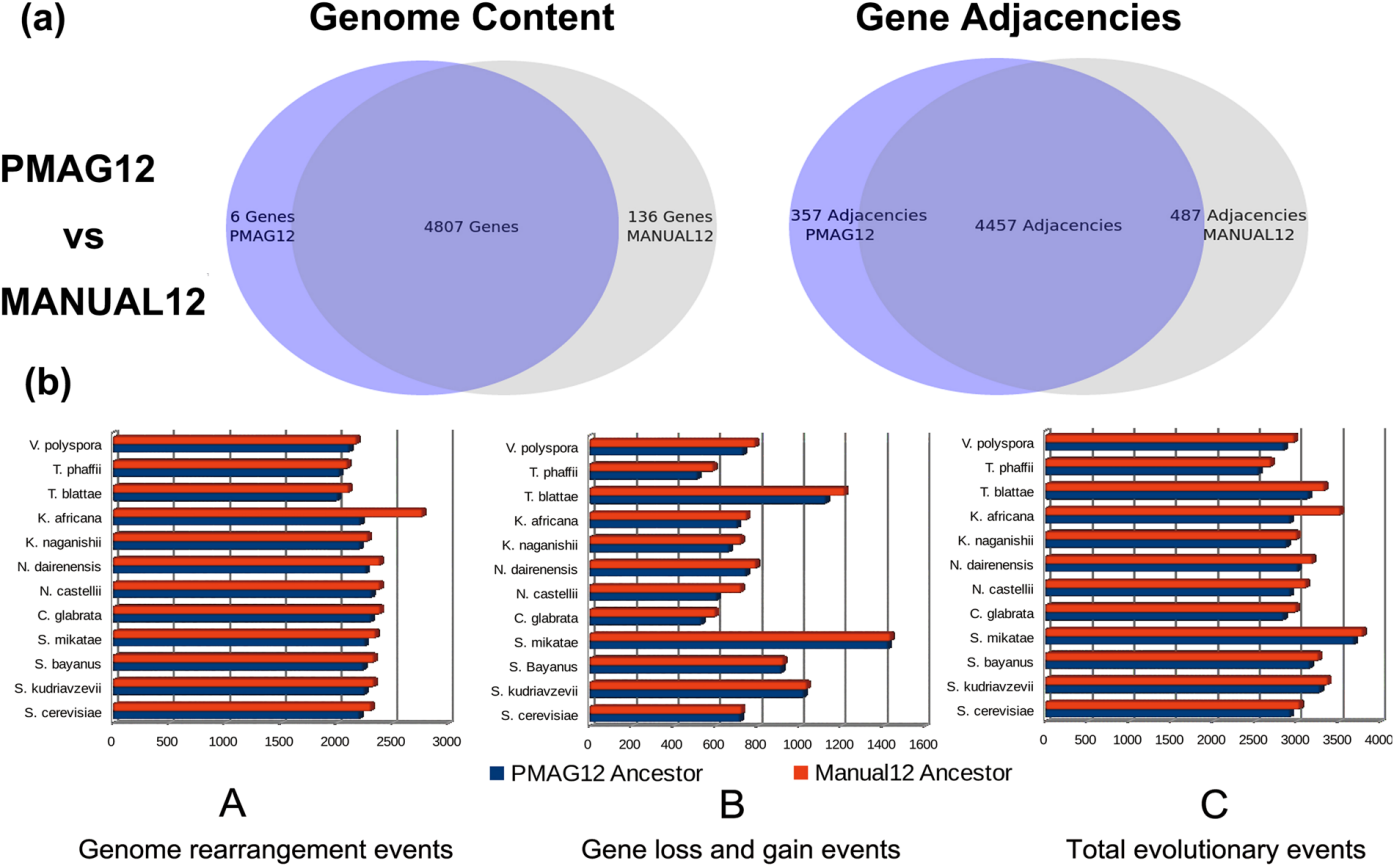
(**a**) Genome content and gene adjacency comparisons between PMAG12 and MANUAL12 ancestors. (**b**) Evolutionary events comparisons between the evolutionary histories of PMAG12 and MANUAL12 ancestors. Figure (**b**) (A–C) show the total number of genome rearrangements events, gene loss and gain events, and overall evolutionary events between ancestral genomes and their shared 12 present post-WGD descendants.

We further reconstructed the ancestral genomes for the second yeast genome dataset with 20 species. We used the phylogeny reconstructed from the same input data in Fig. 1(b) as the guide tree, and reconstructed the ancestral genomes in 55 minutes. There are 18 ancestral genomes reconstructed and labeled as A1–A19 in the phylogeny of Fig. 1(b). Gene numbers of each ancestral genome vary between 4,750 and 5,122. We listed the ancestral genes for each ancestor, and analyzed their functions in Supplementary Dataset 2. Among the 18 ancestral genomes reconstructed here, we use ‘PMAG12’ (also labeled as A18 in Fig. 1(b)) to represent the pre-WGD ancestor at the same evolutionary step with MANUAL12. The post-WGD ancestor A17 had an additional copy of pre-WGD ancestor A18, therefore, it ad the same gene orders and genome information as pre-WGD ancestor A18. There are 4,813 genes in PMAG12 and 4,943 genes in MANUAL12. As Fig. 3(a) shows, these two ancestors are extremely similar. PMAG12 shares 4,807 (99.9%) of its genes and 4,457 (92.6%) of its gene adjacencies with MANUAL12. We compared the different reconstructions on the proportions of adjacencies that were split during evolution. There were 269 “non-split adjacencies” shared by the descendant genomes of PMAG12 and MANUAL12. In our reconstructed ancestral genomes, PMAG12 contained 258 (5.3%) “non-split adjacencies”. MANUAL12 contained 253 (5.1%) “non-split adjacencies”. After removing all “non-split adjacencies”, PMAG12 still shared 4,199 (92.1%) adjacencies with the “benchmark ancestral genome” MANUAL12. We continued to compare the number of different types of evolutionary events between ancestors and their 12 descendants. As Fig. 3(b) shows, even though PMAG12 and MANUAL12 are built from the same dataset, PAMG12 has fewer genome rearrangements, fewer gene losses and gains, and fewer total evolutionary events than MANUAL12 in their evolutionary history.

These results also illustrate that our ancestors are very similar to the manually reconstructed ancestors in genome content and gene adjacencies. Furthermore, our automated approach searches for globally optimal gene permutations with maximum likelihood across the entire genome. However, the manual approach only scans a fixed number of genes, and visually compares these gene orders^42–44^. Therefore, the manual ancestral genomes might be restricted in a local view of gene adjacency and content, and reach a solution with a set of local optimums. Moreover, as we expected, Fig. 3(a) shows that PMAG12 is more similar to the latest ‘benchmark’ ancestor MANUAL12 when compared with our PMAG09 built from the first dataset in Fig. 2(a).

### Ancestral genome reconstruction on simulated data

Currently, only a few computational ancestral reconstruction approaches can process all kinds of evolutionary events and non-identical content genomes. ANGES^38^, Gapped Adjacency^26^, and MGRA2^16^ are reported to be capable of handling all kinds of genome level evolutionary events. However, ANGES can only process low-resolution datasets^34,38^. In this study, we considered each single gene as a marker to reach the highest resolution. We compared our approach with Gapped Adjacency and MGRA2, and evaluated their performances. We simulated a series of large-scale non-identical content yeast datasets with different types of events. In order to make the simulated data more close to the actual genome data, we statistically analyzed the evolutionary rates of current yeast species, and incorporated the rates of different types of events into the simulator.

We simulated each dataset with 20 genomes. The start genome contained eight chromosomes and 5,000 genes. The number of gene adjacencies changes per edge along the phylogeny was set in the interval of [2750, 8250], which kept the “non-split adjacencies” at an extremely low rate. In addition, we calculated the “non-split adjacencies” that shared by all simulated datasets. There were only five “non-split adjacencies” on average in each simulated datasets. So the “non-split adjacencies” was not an issue for the simulated dataset, since there were only few of them under this high evolutionary rate.

To make these experiments statistically reliable, we simulated 10 independent datasets with distinct phylogenies. For each dataset, we needed to reconstruct 18 ancestral genomes, including 1 root ancestor and 17 internal ancestors. We compared the overall average accuracies and running time in reconstructing the genome content and gene adjacencies. The performances of ancestral genomes reconstruction were evaluated by the rate of correctly reconstructed genes and gene adjacencies divided by the total number of genes and gene adjacencies) in both reconstructed and true ancestors: $\tfrac{(G\cup G^{\prime} )}{(G\cap G^{\prime} )}$. G and G′ represented the genes and gene adjacencies in the reconstructed and true ancestral genomes. There were two types of errors: false positive (FP) and false negative (FN). False positives were genes and gene adjacencies were existing in G but missing in G′. The false negatives were defined similarly, by swapping G and G′.

For ancestral genome content reconstruction, our approach PMAG can achieve a very high overall average accuracy of 99.7%. Gapped Adjacency can reach a lower overall average accuracy of 94.0%. However, MGRA2 cannot produce any output after running for 48 hours (MGRA2 does produce accurate outputs for some simple datasets with identical genome content, smaller set of genes, and low evolutionary rates). For ancestral adjacency reconstruction, PMAG can still maintain a very high overall average accuracy of 95.1%. Gapped Adjacency can only achieve an overall average accuracy of 76.8%, which is not reliable enough for current computational methods. MGRA2 cannot produce any output. For each experiment, PMAG requires 11,530 seconds on average to reconstruct all ancestral genomes, and Gapped Adjacency requires 3,553 seconds on average to complete the same task.

In conclusion, out method PMAG outperforms Gapped Adjacency and MGRA2 in the accuracies of ancestral genome contents and adjacencies reconstructions. Both PMAG and Gapped Adjacency can achieve a very high accuracy in reconstructing genome content. However, Gapped Adjacency cannot reconstruct reliable genome adjacencies for ancestral genomes, because its accuracy is under 80%. Additionally, Gapped Adjacency requires less running time than PMAG, and MGAR2 cannot produce any output after running for 48 hours for all experiments.

### Evolutionary and functional analyses of syntenic blocks

**Figure 4:**
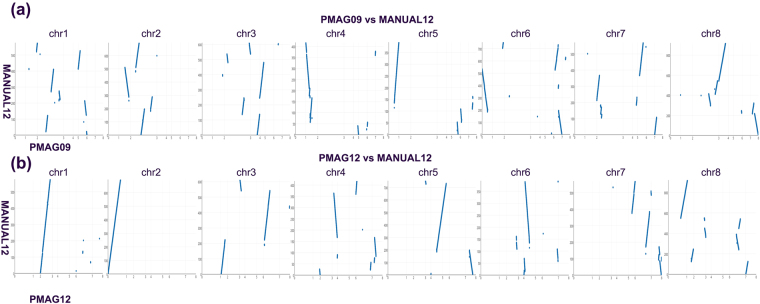
Chromosome dot plots between our ancestors and the “benchmark” ancestor MANUAL12. The eight y-axes of eight sub-figures showed the eight chromosomes of MANUAL12. The x-axes of each sub-figures represented all eight chromosomes of our ancestors.

Comparisons analyses of syntenic blocks between genomes are powerful approaches to study genomic evolution, gene origin, and gene co-evolution^15,50^. In this study, we followed the rigorous and precise rules to define the syntenic blocks, which was the genomic regions that contain two or more genes, maintaining the same gene order and orientation^51^. We ran whole genome comparisons among our automated reconstructed ancestors and manually reconstructed ancestors. We identified all syntenic blocks and analyzed their gene functions in Supplementary Dataset 3. As Table 1 shows, PMAG09 shares 337 syntenic blocks with MANUAL09, which contains 4553 (93.7%) syntenic genes. PMAG09 shares 256 syntenic blocks with MANUAL12, which contains 4753 (94.2%) syntenic genes. Although both PMAG09 and MANUAL09 are reconstructed from the same dataset, PMAG09 is more similar to MANUAL12 in genome contents and chromosome sub-structures (shares more syntenic genes, less syntenic blocks, and longer syntenic block lengths). Table 1 also shows that PMAG12 shares 233 syntenic blocks with MANUAL12, which contains 4733 (93.7%) syntenic genes. PMAG12 has the highest similarity with MANUAL12 in genome contents and sub-structures when compared with MANUAL09 and PMAG09. We also draw the chromosome dot plots between our ancestors and the “benchmark” ancestor MANUAL12, which could illustrate the level of colinearity between them. As the Fig. 4(a,b) shown, our reconstructed ancestral genome PMAG09 and PMAG12 have good chromosome colinearity with the “benchmark” ancestral genome MANUAL12, which illustrated that they shared many chromosome sub-structures. In addition, Fig. 4(b) also illustrates that our PMAG12 ancestor has better chromosome colinearity with the “benchmark” ancestral genome MANUAL12 when compared with PMAG09.

**Table 1:** Syntenic genes and blocks among ancestral genomes.

Manual Ancestral Genomes	PMAG09 Ancestor			PMAG12 Ancestor		
	Genes	Blocks	Avg Length	Genes	Blocks	Avg Length
MANUAL09 Ancestor	4553	337	13.51	4554	319	14.28
MANUAL12 Ancestor	4753	256	18.57	4733	233	20.31

As we expected, our ancestor PMAG09 and PMAG12 agree more with the latest ‘benchmark’ ancestor MANUAL12 in genome contents and structures than ancestor MANUAL09 that build from the first dataset. Furthermore, our PMAG12 ancestor agrees more with MANUAL12 than our PMAG09 ancestor, since the reconstruction of PMAG12 uses additional information. The above results are also consistent with the results that we obtained in the ancestral genome dot plots in Fig. 4, as well as the results in the ancestor genome contents and gene adjacencies comparisons in Figs 2(a) and 3(a).

Next, we continued to run whole genome comparisons between all automated/manually reconstructed ancestral genomes and their five present post-WGD descendants to identify their shared conserved syntenic blocks. Table 2 shows that both PMAG09 and PMAG12 share more syntenic genes with the present yeast species than MANUAL09 and MANUAL12 do. They also preserve less syntenic blocks and longer average syntenic block lengths with present yeast species. These results demonstrate that our ancestors PMAG09 and PMAG12 share more genome contents and sub-structures with the present yeast species when compared with manually reconstructed ancestors. These results also agree with our previous results in Figs 2(b) and 3(b). Furthermore, we annotated the genes and analyzed their functions for each syntenic blocks among automated/manually reconstructed ancestors and present yeast species in Supplementary Dataset 4. This information is critical to locating conserved co-evolution genes and functional gene groups that exist in both ancestral and present species. It can be used to discover the correlations between genome level structural and functional variations in yeasts’ evolutionary history.

**Table 2:** Syntenic genes and blocks between ancestral genomes and present yeast genomes.

Present Yeast Genomes	PMAG09			PMAG12			MANUAL09			MANUAL12		
	Genes	Blocks	Avg Length	Genes	Blocks	Avg Length	Genes	Blocks	Avg Length	Genes	Blocks	Avg Length
S. cerevisiae	3524	1123	3.14	3485	1133	3.08	3307	1108	2.99	3474	1152	3.02
S. uvarum	3459	1123	3.08	3411	1125	3.03	3263	1102	2.96	3401	1140	2.98
C. glabrata	3312	1122	2.95	3282	1116	2.94	3094	1072	2.89	3222	1114	2.89
N. castellii	3338	1112	3.00	3323	1111	2.99	3180	1089	2.92	3324	1130	2.94
V. polyspora	3520	1102	3.19	3462	1088	3.18	3315	1065	3.11	3434	1108	3.10

## Discussion

Initially, studies of yeast phylogenies were based on morphological phenotypes and characters such as sexual states, germinations, and fermentations^52^. Currently, widely accepted yeasts phylogenetic approaches are based on multiple sequence alignment, however, still have limitations and often have conflicting results with each other^4–6,8^. In this study, we reconstructed the phylogenies for two high-resolution yeasts genome datasets using the phylogenetic signals from genome level evolutionary events. The comparisons with the NCBI taxonomy and recent publications demonstrated that our approach could also reconstruct very accurate and robust phylogenies. We provide a new and alternative method to resolve the same phylogenetic problems, but use different types of data and phylogenetic signals. Our approach considers each gene as a single marker and uses 14,101 total markers. Therefore, it will not miss the phylogenetic signals from small scale evolutionary events. It skips the multiple sequence alignment step, and avoids conflicting phylogenetic signals from distinct molecular sequences in traditional phylogenetic approaches. Therefore, our approach can eliminate the conflicting issues that exist in current multiple sequence alignment-based phylogenetic approaches. Current whole genome level phylogenetic studies on real data are limited in simplified identical content genomes^35^ and mitochondrial datasets^36,37^. However, our approach uses a new evolutionary model that can process the real whole genome data, non-identical content data, and all types of complex evolutionary events. This model is based on the principle of double-cut-and-join (DCJ) operations, and incorporates the evolutionary rates of the Saccharomycetaceae family. It can be extended to other multiple chromosome species, after we encoding all existing genes into gene orders and marking out all possible homologous genes that shared by more than one genomes.

Reconstructing ancestral genomes offers opportunities to study the evolutionary mechanisms and trajectories of present species. Studies that focused on developing computational ancestor reconstruction approaches face many difficulties. Present computational approaches suffer from issues of simplistic evolutionary models, complex datasets, and complex evolutionary events^14,15,17,18,25,28,34^. Recent studies of Vakirlis’ *et al*. have made great achievements on computational ancestral reconstruction approaches. They newly sequenced ten yeast species in the Lachancea genus and used software AnChro to reconstruct the ancestral genomes on the Lachancea genus level^5^. They used SynChro to identify conserved syntenic blocks based on the DNA sequence information and additional parameters^53^. They used ReChro to identify cycles of breakpoints for each pairwise combination of genomes^54^. They provided a granular view of genome evolution within an entire eukaryotic genus^5^. In this study, we built an automated pipeline to reconstruct phylogenies and ancestral genomes from whole genome data. First, we built the phylogenies, and then used them as guide trees to reconstruct ancestral genomes from the same input data. Experiments on simulated datasets illustrate that our approach achieves better performances than other current, automated ancestor reconstruction approaches. Ancestor reconstructions on two real yeast genome datasets show that our ancestors are very similar to the manually reconstructed “benchmark” ancestral genomes in genome contents, gene adjacencies. They also shared many chromosome sub-structures with each other. Our maximum likelihood based ancestral reconstruction approach treats the entire genome as a single stage out of billions of possible genome permutation stages, and searches for the global optimal genome permutations. We considered each gene as a basic marker, and reconstructed ancestral genomes by maximizing the overall conditional probabilities of the ancestral marker sets along the edges of phylogenetic tree. We used a TSP (Traveling Salesman Problem) solver to connect ancestral gene adjacencies into genomes with maximum probability, which could successfully assemble ancestral genes into complete chromosomes with low error rates.

Whole genome level evolutionary studies improve our understanding of evolutionary procedures, gene origins, and gene co-evolutions^15,20^. Studies of syntenic blocks in gene order level have several applications in analyzing the genome’s structural and functional evolutions^15^. In this study, we identified the syntenic blocks, which were shared by our ancestors and manually reconstructed ancestors. Analysis results of the syntenic blocks can obtain the same conclusions with the comparison results from genome content and adjacencies discussed in Figs 2 and 3. In Supplementary Dataset 3, we provided functional analyses of these conserved syntenic genes and blocks. The information from these ancestral syntenic blocks is more reliable and credible since it is confirmed by two different ancestral reconstruction approaches. We also identified the conserved syntenic blocks between the ancestral genomes and their shared five present post-WGD descendants. In Supplementary Dataset 4, we provided functional analyses for them. These results can be used to locate the syntenic genes and blocks, which inherited from the same ancestor to maintain their functional relationship during evolutionary processes. Genome level evolutionary events may also break old syntenic blocks, and bring new blocks with new genetic relationships, which can result in structural and functional variation. By analyzing the correlations between evolutionary events and these functional variations, we can use syntenic blocks as genome markers to detect critical evolutionary events, such as functional gene gain and loss. By combining the information on ancestral genomes found in Supplementary Datasets 1 and 2, we can study the genotypes and phenotypes of ancestral genomes that have gone extinct. Annotated gene orders within shared syntenic blocks can be used to locate the orthologous genes across different species. We can use them to trace the gene origins, evolutionary paths, and functional variations in yeasts’ evolutionary histories.

## Methods

### Yeast genome datasets

We reconstructed phylogenies and ancestral genomes for two yeast whole genome datasets. Both datasets are available in the Yeast Gene Order Browser (YGOB) (http://ygob.ucd.ie)^42^. The first dataset contains the genome data of 11 yeast species (Version 3, April 2009), including five post-WGD species under four genera (*S*. *cerevisiae*, *S*. *uvarum*, *C*. *glabrata*, *N*. *castelliie*, *V*. *polyspora*) and six non-WGD species under four genera (*Z*. *rouxii*, *K*. *lactis*, *E*. *gossypii*, *L*. *kluyveri*, *L*. *thermotolerans and L*. *waltii*). This is the same dataset that used in Gordon’s study for reconstructing yeast ancestor MANUAL09^42,43^. The second yeast dataset is also available in YGOB (Version 7, August 2012), and contains nine additional species compared to the first dataset^42,44^. Twelve species are post-WGD species under six genera (*S*. *uvarum*, *S*. *kudriavzevii*, *S*. *mikatae*, *S*. *cerevisiae*, *V*. *polyspora*, *T*. *phaffii*, *T*. *blattae*, *N*. *dairenensis*, *N*. *castellii*, *K*. *naganishii*, *K*. *africana*, *C*. *glabrata*). Eight species are non-WGD species under five genera (*Z*. *rouxii*, *T*. *delbrueckii*, *K*. *lactis*, *E*. *gossypii*, *E*. *cymbalariae*, *L*. *kluyveri*, *L*. *thermotolerans*, *L*. *waltii*). This is also the same data used by Byrne and Wolfe to reconstruct the ‘benchmark’ version of ancestral genome MANUAL12 in the latest version of YGOB^42,44^.

### Binary encoding yeast genome data

In this study, we used gene orders to represent gene permutations and directions on the chromosomes of yeast genomes. We considered each single gene as a genome marker, and used distinct integers to represent the homologous genes across different yeast species. The yeasts homologous genes were defined by the original database YGOB, which was based on the BDBH BLASTP (E < 1e^−5^) using *L*. *waltii* and *S*. *cerevisiae* as reference genomes^42^. Each group of homologous genes was represented by a specific gene order, no matter how many genes were in this group. We used corresponding gene orders to represent the gene permutations and positioning relationships on yeasts genomes for all species. In this study, there were total 14,101 gene orders in our high-resolution genome marker set that used for phylogeny and ancestral reconstructions. The sign (+/−) of a gene order indicates gene’s direction or strand. Each gene is labeled by two ends, head and tail. The head represents a gene’s 5′ end, and the tail represents the 3′ end. For example, gene 1 can be represented by {1*h*, 1*t*}. We used the gene ends and their adjacencies to describe the permutations and positional relationships for all genes on the chromosome. For instance, if gene 1 and gene 2 were adjacent, or gene −2 was followed by gene −1 equivalently, then these two genes can form a gene adjacency {1*t*, 2*h*}. A gene order sequence {1, −2, 3, 4} can be labeled by a set of gene adjacencies: {1*t*, 2*t*}, {2*h*, 3*h*}, {3*t*, 4*h*}. In this paper, our algorithms further encoded genome content and gene adjacencies into binary sequences for each chromosome. For instance, for two genomes with only one chromosome, G1 = {1, −2, 3, 4}, and G2 = {1, 2, 3, 4, −5}, we binary encode them as shown in Table 3.

**Table 3:** Binary encoding genome data.

Genomes	Gene Adjacencies						Genome Content				
	{1*t*, 2*t*}	{1*t*, 2*h*}	{2*t*, 3*h*}	{2*h*, 3*h*}	{3*t*, 4*h*}	{4*t*, 5*t*}	1	2	3	4	5
Genome 1	1	0	0	1	1	0	1	1	1	1	0
Genome 2	0	1	1	0	1	1	1	1	1	1	1

### Improving MLWD for yeasts phylogenies reconstruction

The previous MLWD method was restricted by its fixed evolutionary model and the limitations in handling complex evolutionary events, such as deletion, duplication and whole genome duplication. In this study, we considered each single gene as the smallest genome marker to process high-resolution genome datasets. First, we statistically analyzed the evolutionary rates of different types of events for all species under the Saccharomycetaceae family. Next, we calculated the gene content and gene adjacencies transition probabilities according to these genome evolutionary events. Next, we used these transition probabilities to build a constrained evolutionary model based on the principle of double-cut-and-join (DCJ) operation^48,49^. This yeast evolutionary model takes all kinds of genome level evolutionary events into account, including rearrangements, insertions, deletions, and duplications. Based on the DCJ operation, each event will always remove two old adjacencies randomly, and use the new ends to create two new adjacencies. However, our yeast evolutionary model considers that each genome has n genes and *n* + *O*(*C*) adjacencies with constrained adjacency variations. There are $$(\begin{array}{c}2n+2\\ 2\end{array})$$ possible ends. The transition probability to lose an adjacency is estimated by $\tfrac{\mathrm{2(}R+D+I+d)}{n+O(C)}$. The probability to gain a new adjacency is estimated by $\tfrac{\mathrm{2(}R+D+I+d)}{2{n}^{2}+O(n)}$. *n* and *C* represent the total number of genes and chromosomes for a specific species. *R*, *D*, *I* and *d* represent the estimated number of rearrangements, duplication, insertion, and deletion events for this species based on the evolutionary rates of Saccharomycetaceae family. We also applied their corresponding transition probabilities to the ancestral genome reconstructions. After encoding the yeast genomes into binary sequences (as shown in Table 3) and computing the transition probabilities, we fed this information into the phylogeny reconstruction program RAxML with fast bootstrapping^55^ to reconstruct the yeast phylogeny with overall maximum likelihood. The reconstructed phylogeny and the same input genome data will be fed into the next stage PMAG for ancestral reconstruction. The bootstrapping support value for each internal node and leaf on the phylogeny is considered in three levels: strong support (bootstrap value > 90), medium support (bootstrap value between 60 and 90), and weak support (bootstrap value < 60).

### Improving PMAG for yeasts ancestral genomes reconstruction

Most present computational ancestral reconstruction approaches can only process simplified real data or simulated datasets with unique genome marker and limited types of evolutionary events^14,17,18,30–33^. The previous version of our PMAG approach also faced the same problems^23,32^. In this study, we improved the algorithms of PMAG approach to process all kinds of evolutionary events and high-resolution real genome data. Our improved approach was based on Bayes theorem and probabilistic frameworks. It used the yeast genome transition evolutionary model to compute the gene adjacencies of a specific ancestral genome for each edge of the yeast phylogeny. We considered each gene as a basic genome marker, and reconstructed the ancestral genomes by maximizing the overall conditional probabilities of ancestral marker sets along the edges of the phylogenetic tree.

First, we encoded genome content and gene adjacencies into binary sequences, as shown in Table 3. The duplicated genes and adjacencies were encoded as additional distinct elements, and stored into an additional matrix. We further computed the conditional probabilities for all possible adjacencies across all genomes, and assigned the probabilities as their weights. Next, we used the reconstructed yeast phylogeny (from the phylogeny reconstruction step) as the guide tree, and assembled gene adjacencies into ancestral genomes for all internal tree nodes with the maximum portability. The existence of gene or adjacency in ancestral genome is determined by its conditional probability in the present state. Suppose *a* is the ancestral node on a phylogenetic tree. The conditional probability of node *a* that it has a gene/adjacency *G* is *P*(*G*_*a*_|*O*_*a*_), representing in Equation (1):

1 $$P({G}_{a}|{O}_{a})=\frac{P({G}_{a})\,P({O}_{a}|{G}_{a})}{P({O}_{a})}=\frac{{f}_{{G}_{a}}{p}_{a}({G}_{a})}{{\sum }_{{G}_{a}}\,{f}_{{G}_{a}}{p}_{a}({G}_{a})}$$

*O*_*a*_ is currently observed state in all the subtrees of the ancestral node *a*. ${f}_{{G}_{a}}$ is the frequency of a gene/adjacency *G*_*a*_. *p*_*a*_(*G*_*a*_) is the observed probability of leaves in the subtree of node *a*, which has the gene/adjacency *G*. It can further be calculated by Equation (2):

2 $${p}_{a}({G}_{a})={\sum }_{{G}_{r}}\,{T}_{{G}_{a}{G}_{r}}({d}_{r}){p}_{r}({G}_{r})\times {\sum }_{{G}_{l}}\,{T}_{{G}_{a}{G}_{l}}({d}_{l}){p}_{l}({G}_{l})$$

*r* and *l* represent the left and right children of the ancestor node *a*. *T*_*ab*_(*d*) is the transition probability of gene *a* changing to gene *b* after *d* steps evolution.

The reconstruction of each ancestral genome was not only based on the genome information of its descendants, but also based all available genomes in the datasets. For each ancestral genome reconstruction, we re-rooted the tree and treated the ancestral genome that needed to reconstruct as the new root, and built the ancestral genome with the global maximum probability over all species. We reconstructed gene orders of ancestral nodes by using a similar idea from a previous probabilistic reconstruction approach for sequence data^56^. We also used the RAxML^55^ to estimate evolutionary distance *d*. Our method iterated the steps above to compute all of probabilities of adjacencies for each internal genome. Once all of these probabilities were obtained, We converted this genome adjacencies assembly/reconstruction problem into an instance of the Traveling Salesman Problem (TSP), and used the Chained-Lin-Kernighan heuristic TSP solver Linkern to solve this problem^57^. The original yeasts input genomes were not well assembled. Many genome contigs couldn’t be assembled or mapped back to regular chromosomes. Even though the real yeast genome data suffered from these issues, the TSP solver helped us to better assemble/reconstruct ancestor genomes by using all available adjacencies information in known genomes. We mapped the gene content, gene adjacencies, and duplicated genes information with the outputs from TSP solver. Finally, we determined the chromosome structures of reconstructed ancestral genomes based on the chromosome structure information of its parent and children genomes. We first identified all chromosome telomeres for its parent and two children genomes. Next, we introduced the ancestral chromosome telomeres with new chromosome telomeres by the following orders: 1, telomeres shared by all three genomes (one parent and two children genomes); 2, shared by two children genomes; 3, shared by any two genomes; 4 existed in the parent genomes; 5 existed in one of children genomes. Finally, when the chromosome number reached the number of its two children genome or its parent genome, we output the updated ancestral genomes with correct chromosome number and chromosome telomeres.

### Code Availability

Code package is available in the Supplementary Info files. There is a ReadMe.txt file to show how to configure and run the package.

## Electronic supplementary material

Supplementary Documents

Supplementary Dataset 1

Supplementary Dataset 2

Supplementary Dataset 3

Supplementary Dataset 4
