# Supplementary material for: Reconstructing Yeasts Phylogenies and Ancestors from Whole Genome Data
Source: Sci Rep. 2017 Nov 9;7:15209. doi: 10.1038/s41598-017-15484-5 (PMC5680185; doi:10.1038/s41598-017-15484-5)
Supplement: Supplementary file 1 — Supplementary Documents [file 41598_2017_15484_MOESM1_ESM.pdf]

Supplementary Documents:
Reconstructing Yeasts Phylogenies and Ancestors from Whole Genome Data

Bing Feng[1,2], Yu Lin[3], Lingxi Zhou[2,4], Yan Guo[6], Robert Friedman[5], Ruofan Xia[2], Fei Hu[2] , Chao Liu[1,*], and Jijun Tang[2,*]

[1]Institute of Education, Tsinghua University, Beijing, 100084 PR China

[2]Department of Computer Science and Engineering, University of South Carolina, Columbia, SC 29208, USA

[3]College of Engineering and Computer Science, The Australian National University, Acton, ACT 2601, Australia

[4]School of Computer Science and Technology, Tianjin University, Tianjin 300072, PR China

[5]Department of Biological Science, University of South Carolina, Columbia, SC 29208, USA

[6]Center for Quantitative Sciences, Vanderbilt University, Nashville, TN 37232 USA[*]

jtang@cse.sc.edu and lcpku@163.com

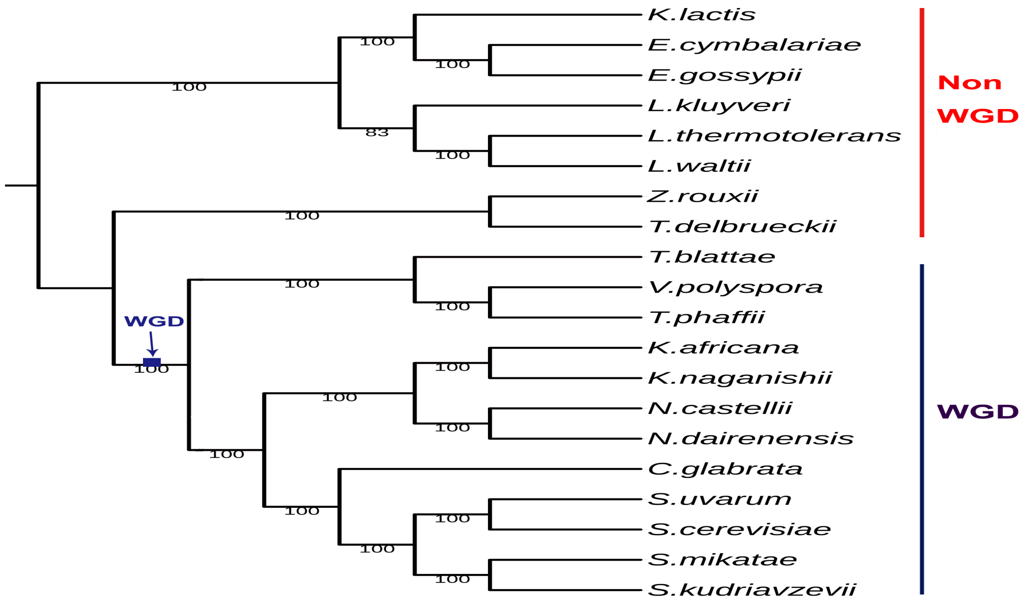

Supplementary Figure1. Yeasts phylogeny built from genome rearrangement events for 20 yeast species. All of the edges have a bootstrapping value of 100 except the branch between Lachancea genus and Eremothecium genus (bootstrapping value of 83). The red line on the right shows the non-WGD species, and the blue line on the right shows the post-WGD species. The blue bar on the left shows the

evolutionary step before yeasts' WGD event.

Code Package Downloading:

Please download the package and running examples using this link:

https://www.dropbox.com/sh/1pr4pbabykz15d9/AAD6_Hp7aKQAcwOjkvVP1liRa?dl=0

Supplementary Datasets 1-4 are provided in extra four xls files.

Supplementary Dataset 1: Ancestral genomes information reconstructed from 11 yeast species.

The reconstructed ancestral genomes information are provided in nine separate sheets.

Supplementary Dataset 2: Ancestral genomes information reconstructed from 20 yeast species.

The reconstructed ancesral genomes information are provided in 18 separate sheets.

Supplementary Dataset 3: Syntenic blocks analyses among ancestral genomes.

The syntenic blocks information among automated and manually reconstructed ancestral genomes are provided in four separate sheets.

Supplementary Dataset 4: Syntenic blocks analyses among ancestral genomes and present yeast genomes.

The syntenic blocks information among automated/manually reconstructed ancestral genomes and their shared five post-WGD descendants are provided in 20 separate sheets.
